# Coordinated reprogramming of renal cancer transcriptome, metabolome and secretome associates with immune tumor infiltration

**DOI:** 10.1186/s12935-022-02845-y

**Published:** 2023-01-05

**Authors:** Piotr Poplawski, Saleh Alseekh, Urszula Jankowska, Bozena Skupien-Rabian, Roksana Iwanicka-Nowicka, Helena Kossowska, Anna Fogtman, Beata Rybicka, Joanna Bogusławska, Anna Adamiok-Ostrowska, Karolina Hanusek, Jan Hanusek, Marta Koblowska, Alisdair R. Fernie, Agnieszka Piekiełko-Witkowska

**Affiliations:** 1 Department of Biochemistry and Molecular Biology, Centre of Postgraduate Medical Education, ul. Marymoncka 99/103, 01-813 Warsaw, Poland; 2 Max-Planck Institute of Molecular Plant Physiology, Golm, 14476 Potsdam, Germany; 3 Center for Plant Systems Biology and Biotechnology, 4000 Plovdiv, Bulgaria; 4 Proteomics and Mass Spectrometry Core Facility, Malopolska Centre of Biotechnology, Jagiellonian University, Kraków, Poland; 5 Laboratory of Systems Biology, Faculty of Biology, University of Warsaw, 02-106 Warsaw, Poland; 6 Laboratory for Microarray Analysis, Institute of Biochemistry and Biophysics, Polish Academy of Sciences, 02-106 Warsaw, Poland

**Keywords:** Renal cancer, SPARC, Secretome, Metabolome, CAFs, Immune infiltration

## Abstract

**Background:**

Clear cell renal cell carcinoma (ccRCC) is the most common subtype of renal cancer. The molecules (proteins, metabolites) secreted by tumors affect their extracellular milieu to support cancer progression. If secreted in amounts detectable in plasma, these molecules can also serve as useful, minimal invasive biomarkers. The knowledge of ccRCC tumor microenvironment is fragmentary. In particular, the links between ccRCC transcriptome and the composition of extracellular milieu are weakly understood. In this study, we hypothesized that ccRCC transcriptome is reprogrammed to support alterations in tumor microenvironment. Therefore, we comprehensively analyzed ccRCC extracellular proteomes and metabolomes as well as transcriptomes of ccRCC cells to find molecules contributing to renal tumor microenvironment.

**Methods:**

Proteomic and metabolomics analysis of conditioned media isolated from normal kidney cells as well as five ccRCC cell lines was performed using mass spectrometry, with the following ELISA validation. Transcriptomic analysis was done using microarray analysis and validated using real-time PCR. Independent transcriptomic and proteomic datasets of ccRCC tumors were used for the analysis of gene and protein expression as well as the level of the immune infiltration.

**Results:**

Renal cancer secretome contained 85 proteins detectable in human plasma, consistently altered in all five tested ccRCC cell lines. The top upregulated extracellular proteins included SPARC, STC2, SERPINE1, TGFBI, while downregulated included transferrin and DPP7. The most affected extracellular metabolites were increased 4-hydroxy-proline, succinic acid, cysteine, lactic acid and downregulated glutamine. These changes were associated with altered expression of genes encoding the secreted proteins (SPARC, SERPINE1, STC2, DPP7), membrane transporters (SLC16A4, SLC6A20, ABCA12), and genes involved in protein trafficking and secretion (KIF20A, ANXA3, MIA2, PCSK5, SLC9A3R1, SYTL3, and WNTA7). Analogous expression changes were found in ccRCC tumors. The expression of SPARC predicted the infiltration of ccRCC tumors with endothelial cells. Analysis of the expression of the 85 secretome genes in > 12,000 tumors revealed that SPARC is a PanCancer indicator of cancer-associated fibroblasts’ infiltration.

**Conclusions:**

Transcriptomic reprogramming of ccRCC supports the changes in an extracellular milieu which are associated with immune infiltration. The proteins identified in our study represent valuable cancer biomarkers detectable in plasma.

**Supplementary Information:**

The online version contains supplementary material available at 10.1186/s12935-022-02845-y.

## Background

Renal cell carcinoma (RCC) is the most common subtype of kidney cancers affecting > 400,000 patients annually worldwide [1]. The most common subtype of RCC is clear cell renal cell carcinoma (ccRCC), contributing to 75% of cases [2]. Advanced, metastatic ccRCC (mccRCC) is clinically challenging. Despite broad possibilities of targeted therapies involving inhibitors of mTORC and tyrosine kinases (TKIs), as well as therapies involving inhibitors of immune checkpoints, the prognosis for mRCC patients is poor. Therefore, the knowledge of molecular mechanisms contributing to RCC metastatic progression is crucial for finding novel targeted therapies [2, 3].

The understanding of the composition of molecules secreted by tumors is important for finding novel treatment options and prevention of metastatic disease. Tumors secrete factors that affect their microenvironment (TME) and contribute to the creation of pre-metastatic niches (PMNs), facilitating formation of secondary lesions [4]. The formation of PMNs was confirmed in patients with RCC [4]. The specific factors involved in PMNs’ formation include cytokines, growth factors, enzymes modifying ECM (extracellular matrix) or components of exosomes. Important contributors to TME are the secreted products of cells’ metabolism [5]. In particular, extracellular lactate is drawing attention as a modulator of immune activation, angiogenesis, metastasis, and resistance to therapy [6].

Despite several reports concerning the RCC secretome (e.g. [7, 8]), studies aiming at comprehensive analysis of molecules secreted by RCC cells are missing. Here, we performed proteomic and metabolomic analysis of ccRCC conditioned media, combined with transcriptomic analysis of ccRCC cells to provide a list of molecules secreted by RCC which can be studied as potential diagnostic markers and potential therapeutic targets. We demonstrate that the main alterations in the ccRCC secretome are tightly associated with infiltration by immune and endothelial cells. We found SPARC as a main PanCancer secretory protein linked with the presence of cancer-associated fibroblasts (CAFs) in multiple cancer types.

## Methods

Cell lines: RPTEC/TERT1 (CRL-4031), Caki-1 (HTB-46), 786-O (CRL-1932), A498 (HTB-44) cell lines were obtained from ATCC (American Type Culture Collection) and cultured according to the manufacturer protocol. KIJ265T and KIJ308T cell lines were obtained from Mayo Foundation of Medical Education and Research and cultured as described previously [9].

Collection of Conditioned Media (CM): 10^6^ cells were seeded at 75 cm^2^ flasks. After 24 h cells were rinsed once with PBS and 4 times with DMEM without phenol red supplemented with GLUTAMAX. Next, 15 ml of DMEM without phenol red supplemented with GLUTAMAX was added to 75 cm^2^ flasks. After 24 h CM were collected, centrifuged and frozen at -80 °C for further analyses.

RNA isolation from cell lines: cells were trypsinized, centrifuged (120 *g*, 5 min) and resuspended in PBS. RNA was isolated using GeneMATRIX Universal RNA/miRNA Purification Kit (EURX, Gdansk, Poland) in accordance with manufacturer’s protocol. Briefly, cells suspended in PBS were centrifuged (5 min at 1000 *g*) and 400 μl RL buffer was added. Next, after the suspension was mixed by vortexing and pipetting, 100 μl of Lyse ALL was added, mixed and centrifuged at maximum speed for 2 min. The supernatant was transferred to homogenization spin-column, centrifuged (12,000 *g*, 2 min) and 1.2 volumes of 96% [v/v] ethanol were added to the flow-through, mixed by pipetting and transferred to the RNA binding spin-column and centrifuged (11,000 *g*, 1 min). The supernatant was discarded, column was washed with 500 µl of Wash miRNA and centrifuged (11,000 *g*, 1 min). Washing step was repeated two times, then empty column was centrifuged (11,000 *g*, 1 min). 40 µl of RNase-free water was added and RNA was eluted by centrifugation (11,000 *g*, 2 min). RNA concentration was measured with Nanodrop ND-1000 spectrophotometer. RNA was stored at low temperature freezer.

Protein isolation from CM: Proteins were isolated from 40 ml of CM filtered by Milex GV Low Protein Binding Durapore (PVDF) 0.22 µm (EMD Millipore Corporation. Billerica, MA). After centrifugation on Amicon Ultra-4, Ultracel (Merck KGaA, Darmstadt, Germany) proteins were extracted using 0.1 M Tris–HCl pH 7.5, 2% SDS, 0.1 M DTT buffer.

Extraction of metabolites from CM: 1 ml of 80% methanol in water was added to 0.5 ml of CM, vortexed, centrifuged for 15 min. Supernatant 500 µl and 300 µl aliquots were frozen for further analyses.

Protein concentrations in CM were analyzed using ELISA tests: DSE100 Human Serpin E1/PAI-1 Quantikine ELISA Kit (USA R&D Systems, Inc., Minneapolis, MN), DSP00 Human SPARC Quantikine ELISA Kit (USA R&D Systems, Inc., Minneapolis, MN), NBP2-60603 Human Stanniocalcin 2/STC-2 ELISA Kit (Colorimetric) (Novus Biologicals USA, Centennial, CO), KA0981-Human HSP27 ELISA Kit (Colorimetric) (Abnova, Tajpei, Taiwan), ELH-Trfrn-1 RayBio^®^Human Transferrin ELISA Kit and RayBio^®^ELH-PRDX2-1 Human Peroxiredoxin-2/PRDX2 ELISA Kit (RayBiotech, Inc, Norcross, GA), EH160RB Human DPPII/QPP/DPP7 ELISA Kit (Thermo Fisher Scientific Inc./Life Technologies Corporation, Carlsbad, CA), EHTGFBI TGFBI (BIGH3) Human ELISA Kit (Thermo Fisher Scientific Inc. /Life Technologies Corporation, Carlsbad, CA), MBS7206431 Human Adseverin (SCIN) ELISA Kit (MyBioSource, Inc., San Diego, CA), MBS8803863 Human PLOD2 (Procollagen Lysine-2-Oxoglutarate-5-Dioxygenase 2) ELISA Kit (MyBioSource, Inc., San Diego, CA). The analyses were performed according to the manufacturers’ protocols. The brief summary of ELISAs conditions (including incubation times and temperatures) is presented in Additional file 1: Table S1. The analyses were performed using conditioned media isolated from 3 independent biological experiments per each cell line.

Metabolites were analyzed using L-Lactic Acid/Lactate (LA) Colorimetric Assay Kit E-BC-K044-M (Elabscience, Houston, TX), Cysteine (Cys) Colorimetric Assay Kit E-BC-K352-M (Elabscience, Houston, TX) and Glutamine/Glutamate-Glo™ Assay (Promega, Madison, WI) and Fluorimetric Succinate Assay Kit MAK355 (Sigma-Aldrich, St. Louis, MO). The analysis was performed using conditioned media isolated from 3 independent biological experiments per each cell line.

Reverse transcription and qPCR were performed as described previously [10]. The sequences of the primers used in the study are presented in Additional file 1: Table S2. The expression of RNA, 18S Ribosomal N1 was used for normalization of genes’ expression. qPCR analysis was performed using RNA isolated from 3 independent biological experiments in three replicates per each cell line. The qPCR analysis of gene expression in ccRCC tissues was performed using RNA obtained from the Department of Biochemistry and Molecular Biology Bank of RNA, isolated from tumors (n = 92) and matched-paired non-tumorous control samples (n = 92) under approval of the Local Bioethical Committee of Centre of Postgraduate Medical Education (Approval no. 119/PB/2019). The classification of tumor tissue samples according to TNM Stage and Fuhrman grade is shown in Additional file 1: Table S3. RNA from tissues (1 µg) was reverse transcribed using anchored-oligo(dT)18 and random hexamer primers with Transcriptor First Strand cDNA Synthesis Kit (Roche Diagnostics, Mannheim, Germany) in accordance with manufacturers’ protocol.

Microarray analysis was performed using Affymetrix™ HuGene 2.1 ST Array Strips (Affymetrix, Santa Clara, CA, USA) as earlier described [11]. The analysis was performed using RNA isolated from 3 independent biological experiments per each cell line.

Proteomic analysis: The LC–MS/MS analysis and data processing was performed as earlier described [12] using a nanoHPLC system (UltiMate 3000 RSLCnano System, Thermo Fisher Scientific, Waltham, MA, USA) coupled with a Q Exactive mass spectrometer (Thermo Fisher Scientific, Waltham, MA, USA). The analysis was performed using protein extracts isolated from 3 independent biological experiments.

Metabolomics analysis was performed using GC–MS as earlier described [13]. The analysis was performed using metabolic extracts from 3 independent biological experiments.

Bioinformatic and statistical analysis: All experiments were performed using at least 3 independent biological experiments (i.e. cells that were seeded on independent days). GO analysis was performed using ShinyGO 0.76 [14]. Correlation of gene expression with tumor infiltration by immune cells was performed using Timer [15]. Protein analysis in ccRCC tumors was performed using UALCAN/CPTAC platform [16–18]. Microarray data analysis was performed using Transcriptome Analysis Console (TAC) Software 4.0 (ThermoFisher) [11]). The criteria for selecting DEGs were fold change ≤  − 2.0 or fold change ≥ 2.0 and FDR ≤ 0.01. Statistical analysis was performed using GraphPad Prism and the following tests: Shapiro–Wilk test (normality of data distribution), Wilcoxon matched-pairs signed rank test, t‑test, one‑way ANOVA followed by Dunnett's multiple comparison test. FDR was controlled using Benjamini–Hochberg procedure [19].

## Results

### The secretome of ccRCC cell lines is altered when compared with normal kidney cells

**Fig. 1:**
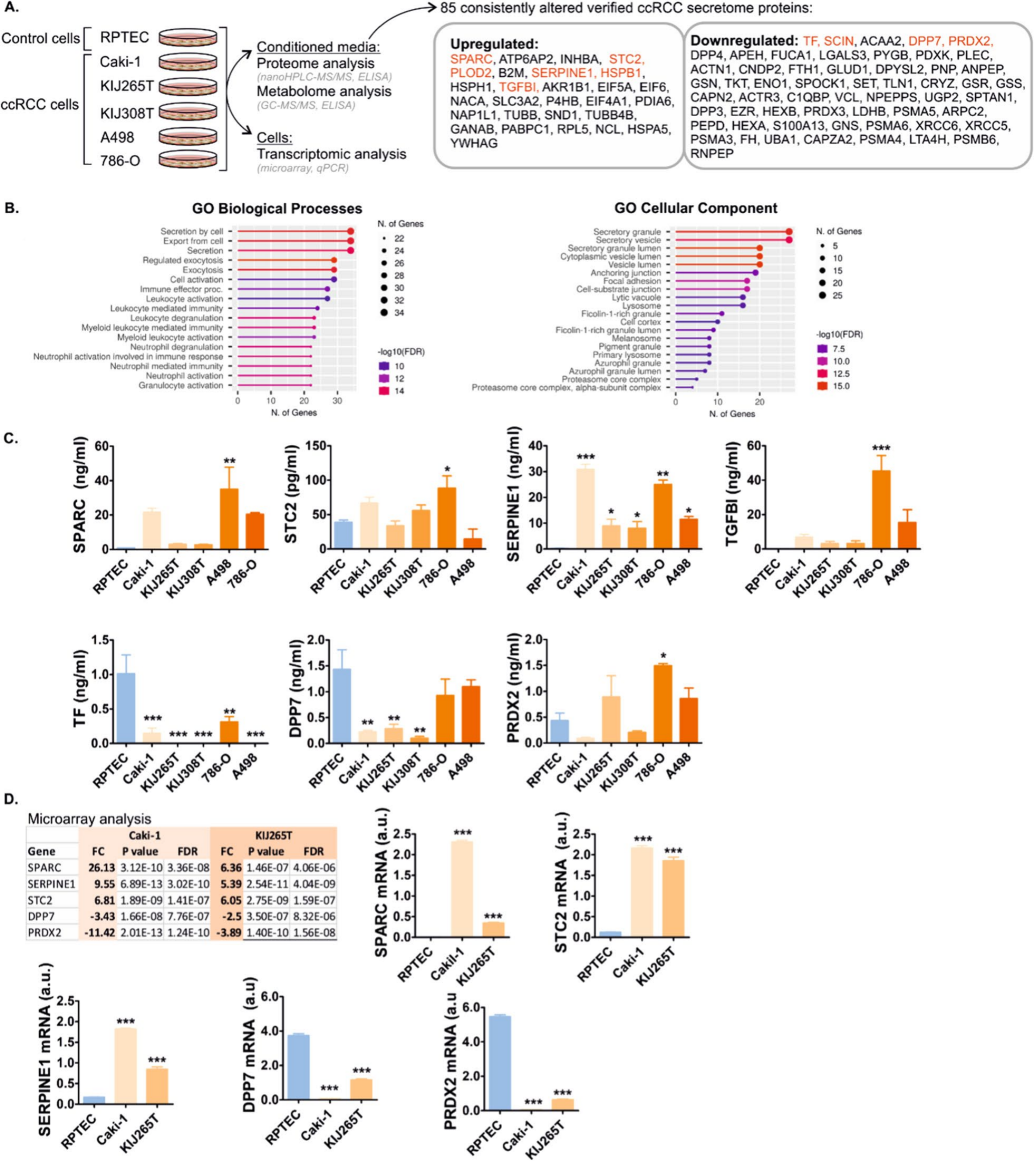
Proteomic analysis of RCC secretome. **A** Left: The scheme of the experiment. CM from all cell lines was subjected to proteomic and metabolomics analysis. Cells were collected and used for transcriptomic analysis. Right: nanoHPLC-MS/MS revealed 85 CM proteins that were consistently altered in all ccRCC cell lines the same direction (upregulated/downregulated) and verified as secretome components. Proteins selected for validation are shown in red font. Complete data are shown in Additional file 1: Table S4. The analysis was performed using protein extracts isolated from 3 independent biological experiments per each cell line. **B** Gene ontology analysis of the 85 consistently altered proteins. **C** Validation of top altered proteins using ELISA. The analysis was performed using conditioned media isolated from 3 independent biological experiments per each cell line. Statistical analysis: One-way ANOVA with Dunnett's Multiple Comparison Test. *p < 0.05, **p < 0.01, ***p < 0.001. **D** The expression of genes encoding altered CM proteins is disturbed in ccRCC cell lines. The table shows result of microarray analysis (complete data are shown in Additional file 1: Table S5). The plots show results of qPCR validation of the analyzed genes in RNA isolated from 3 independent biological experiments. Statistical analysis: One-way ANOVA with Dunnett's Multiple Comparison Test. *p < 0.05, **p < 0.01, ***p < 0.001

When compared with RPTEC, all five RCC-derived cell lines shared 255 aberrantly abundant CM proteins, with 185 proteins of which levels changed in the same direction (Additional file 1: Table S4). To find proteins that were present in CM in actionable amounts, we generated the list of proteins that were expressed in amounts comparable to or higher than MMP1, a well-known secretome component secreted by kidney and non-kidney cells [20–25]. To filter out all proteins that could potentially be by-products of accidental cell damage, we cross-compared these results against the list of proteins previously reported by a transcriptome-based study as RCC secretome [26]. This left us with a strictly restricted list of 85 proteins that were classified as either classical or experimental, and were consistently and uniformly altered in CM from all five analyzed RCC cells lines (Fig. 1A, Additional file 1: Table S4). There were 28 uniformly upregulated proteins and 57 uniformly downregulated proteins. The vast majority of these proteins were qualified as canonical plasma proteins [26] (Fig. 1). GO analysis revealed enrichment of proteins related to cellular secretion, export, exocytosis as well as linked to immunity (e.g. immune effector processes, activation of leukocytes, neutrophils and myeloid leukocytes) (Fig. 1B, Additional file 1: Table S4). The proteins formed a concise interaction network in STRING analysis (Additional file 2: Fig. S1).

Our aim was to identify proteins secreted by ccRCC cell lines that: i) could represent the potential ccRCC biomarkers for future analyses of patients plasma/serum; ii) could represent the molecules that may actively shape the tumor microenvironment. Therefore, to validate the results of proteomic analysis, we selected the most altered proteins with experimentally confirmed associations with ccRCC pathology, cancerous secretion and/or influence on TME (Additional file 1: Table S4) and measured their levels in CM using ELISA. This confirmed uniformly increased levels of SPARC, SERPINE1, and TGFBI, as well as decreased level of TF (transferrin) in CM from RCC cell lines (Fig. 1C). STC2 level was statistically significantly increased in CM from 786-O cells while the level of DPP7 was statistically significantly decreased in CM from three cell lines. PRDX2 had variable levels depending on cell line analyzed. HSP27 (HSPB1) and SCIN did not change in CM from any cell line (Additional file 2: Fig. S2), while PLOD2 was below the limit of ELISA detection.

### Transcriptomic changes reflect altered secretome of RCC cells

**Fig. 2:**
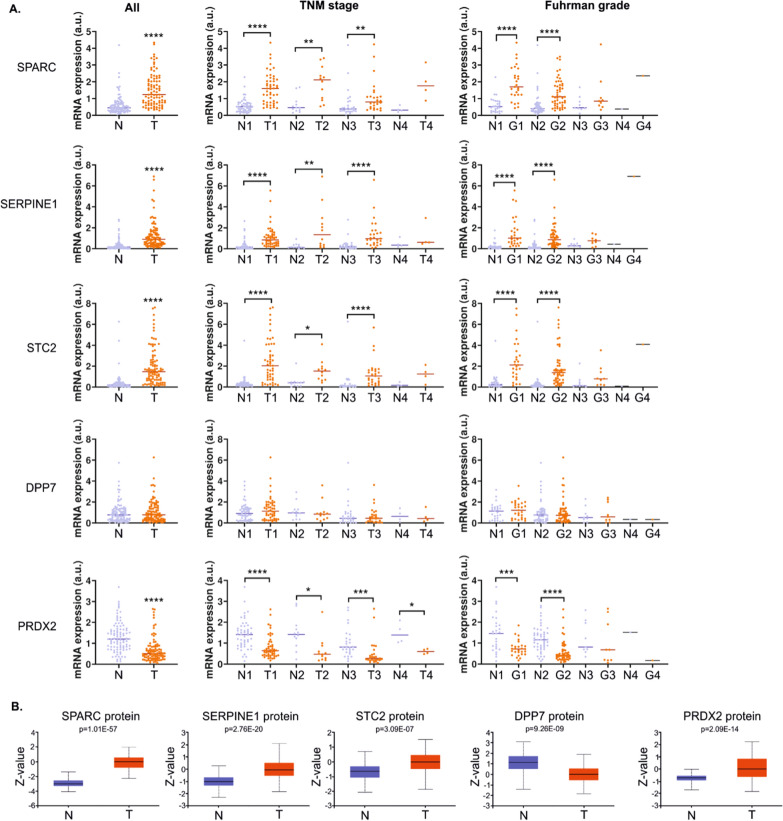
A. The expression of genes encoding proteins of ccRCC secretome is altered in ccRCC tumors. **A** The plots show results of qPCR analysis performed in non-neoplastic kidney samples (N) and ccRCC tumor samples (T). All: analysis performed in all samples, without differentiation into TNM Stage/Fuhrman grade. N: n = 92; T: n = 92; TNM stage: tumor samples were classified into Stage 1 (N1: n = 49, T1: n = 49), Stage 2 (N2: n = 12, T2: n = 12), Stage 3 (N3: n = 27, T3: n = 27), Stage 4: N4: n = 4, T4: n = 4). Fuhrman grade: tumor samples were classified into Grade 1 (N1: n = 27, G1: n = 27), Grade 2 (N1: n = 51, G2: n = 51), Grade 3 (N3: n = 9, G3: n = 8), Grade 4 (N4: n = 1, G4: n = 1). Statistical analysis was performed using Wilcoxon matched-pairs signed rank test or paired t test, depending on data normality distribution. **B** The plots show results of UALCAN/CPTAC analysis on proteomic data from ccRCC tumors (n = 110) and normal kidney tissues (n = 84)

To find the mechanisms behind secretome alterations we analyzed the transcriptome of Caki-1 and KIJ265T cells. There were 1497 genes commonly altered in both analyzed ccRCC cell lines when compared with RPTEC, including SPARC, SERPINE1, STC2, SCIN, DPP7, PRDX2 (Fig. 1D, Additional file 1: Table S5). qPCR validation confirmed altered expression of SPARC, STC2, SERPINE1, DPP7, and PRDX2. Moreover, the expression of these genes and their protein products was also mostly consistently altered in tumors from ccRCC patients (Fig. 2). The exception was DPP7 for which mRNA expression was not altered when analyzed in all tumor tissues, or slightly increased in ccRCC tumors of TNM Stage 1. In contrast to the profile of mRNA expression, PRDX2 protein level was increased in tumors (Fig. 2B).

**Fig. 3:**
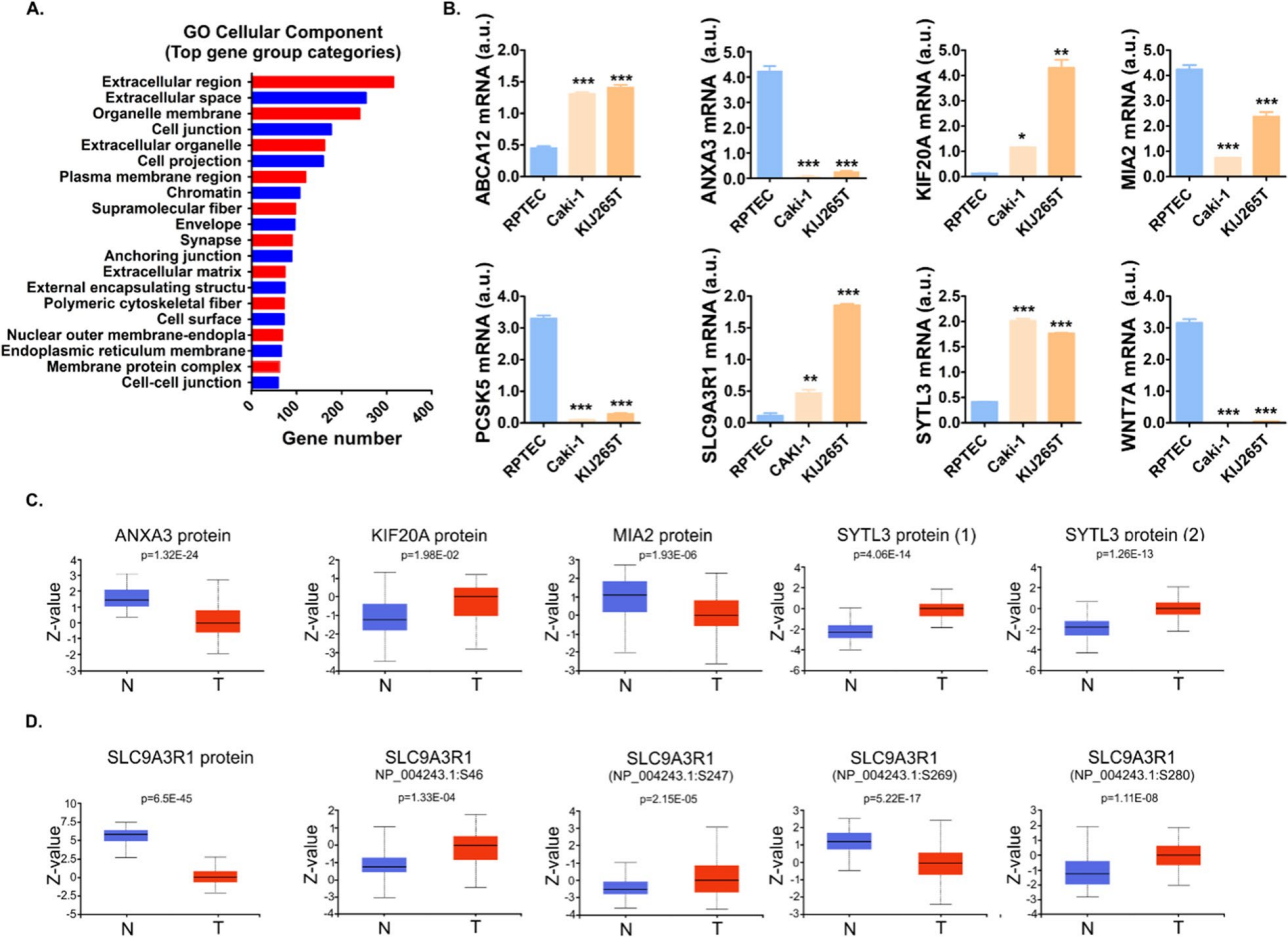
The expression of genes involved in secretion and ECM regulation is altered in ccRCC. **A** GO analysis of 1497 genes commonly altered in Caki-1 and KIJ265T cells when compared with RPTEC. The genes are grouped by functional categories defined by high-level GO Cellular Component terms. The gene group categorization was analyzed using by ShinyGO 0.76 analysis (http://bioinformatics.sdstate.edu/go/) and the data was imported into GraphPad Prism to generate the plot. Complete data are shown in Additional file 1: Table S5. **B** The expression of genes regulating secretion and extracellular space. The plots show qPCR validation of microarray results in RNA isolated from 3 independent biological cell culture experiments. Statistical analysis: One-way ANOVA with Dunnett's Multiple Comparison Test. *p < 0.05, **p < 0.01, ***p < 0.001. **C** The expression of proteins regulating secretion and extracellular space in ccRCC tumors. The plots show results of UALCAN/CPTAC analysis. For SYTL3 only data for phosphopeptides were available (SYTL3 (1): NP_001229313.1:S185. SYTL3(2): NP_001229313.1:S203). N: normal kidney samples (n = 84), T: ccRCC tumors (n = 110). **D** The expression of SLC9A3R1 protein and its phosphorylated variants in ccRCC tumors. N: normal kidney samples (n = 84), T: ccRCC tumors (n = 110). The analysis was performed using UALCAN/CPTAC platform

GO analysis revealed that the major gene group categories expressed by ccRCC cell lines were Extracellular region and Extracellular space (Fig. 3A). The altered genes included those involved in protein transport, secretion and export from the cell (Additional file 1: Tables S5 and S6). In particular, we found aberrant expression of a large group [17] of kinesins required for transport (Additional file 1: Table S5). Among them, the top upregulated was KIF20A, a kinesin crucial for the fission of RAB6-positive vesicles and their exit from Golgi/TGN membranes [27]. qPCR confirmed altered expressions of ABCA12, ANXA3, KIF20A, MIA2, PCSK5, SLC9A3R1, SYTL3, and WNTA7 in ccRCC cell lines (Fig. 3B). Moreover, the expression of genes (Additional file 1: Table S7) and most of the encoded proteins was consistently altered in ccRCC tumors (Fig. 3C). Surprisingly, despite its increased expression in ccRCC cell lines and tumors, SLC9A3R1 protein was downregulated in ccRCC tumors. Since phosphorylation affects SLC9A3R1 functioning [28], we also checked the expression of its phosphorylated variants. Indeed, phosphorylation of SLC9A3R1 was altered in ccRCC tumors (Fig. 3D).

### The genes of ccRCC secretome correlate with immune infiltration

**Fig. 4:**
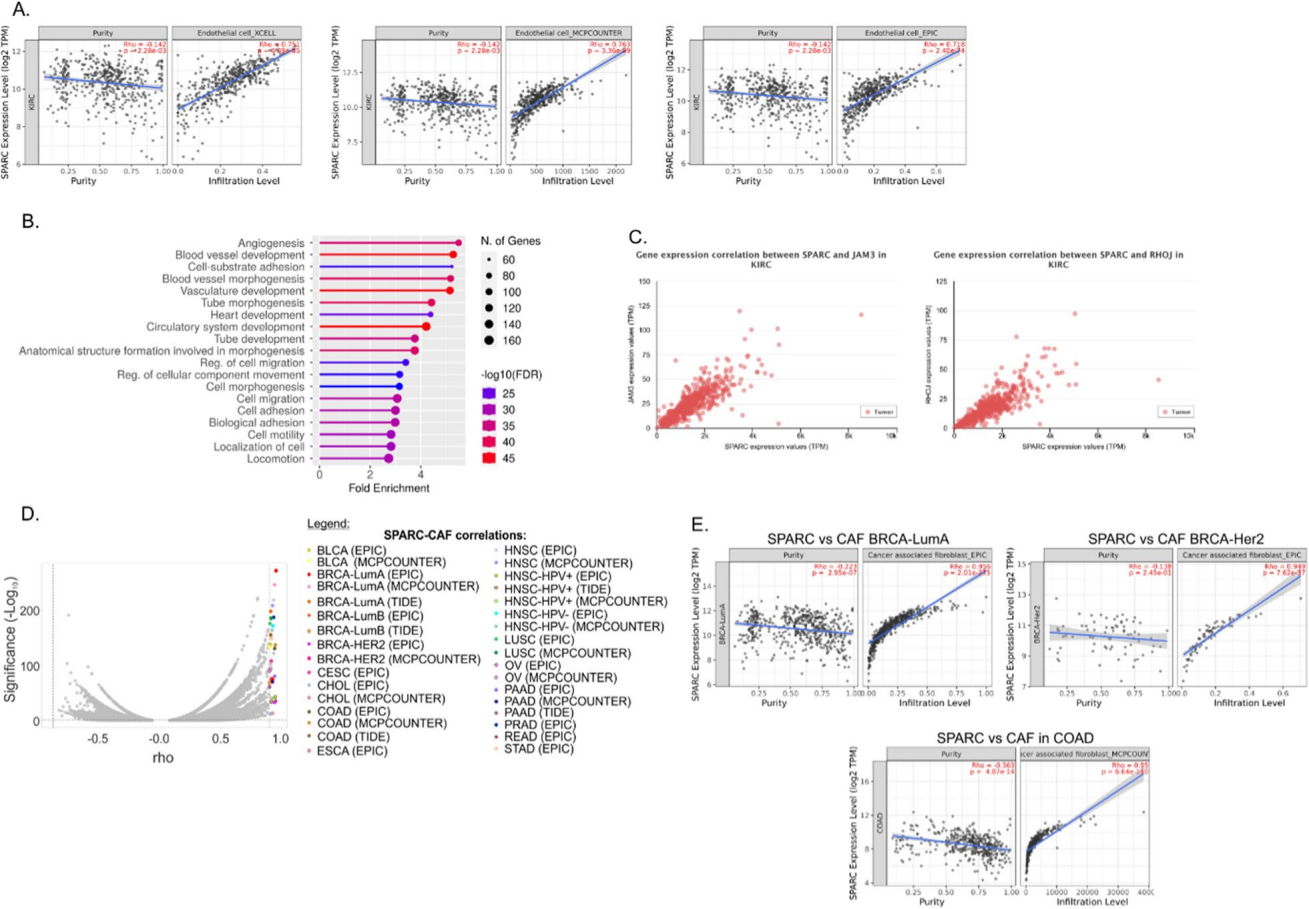
The expression of genes encoding key ccRCC secretome proteins correlates with immune infiltration in tumors. **A** The expressions of SPARC correlates with the presence of endothelial cells in ccRCC tumors. The plots show results of analysis performed with Timer platform (http://timer.comp-genomics.org/). **B** Top enriched terms for biological processes for genes positively correlating with SPARC in ccRCC tumors. Only genes with r ≥ 0.5 correlations were analyzed. The analysis was performed using ShinyGO 0.76 (http://bioinformatics.sdstate.edu/go/). **C** The expression of SPARC correlates with key angiogenic regulators in ccRCC tumors. The plots show results of analysis performed with UALCAN (http://ualcan.path.uab.edu/index.html) platform. **D** SPARC emerges as the top gene correlating with immune infiltration in PanCancer analysis. Volcano plot shows the results of correlation analysis between the expression of 85 genes encoding proteins of ccRCC secretome and the presence of immune cells infiltrating 40 tumor types. SPARC correlations (r ≥ 0.9) are shown with colorful dots. **E** Representative plots showing top SPARC-CAFs correlations in cancers of breast (BRCA-LumA, BRCA-Her2) and colon (COAD). For detailed data see Additional file 2: Table S10. EPIC, MCPCOUNTER, TIDE: different algorithms utilized by Timer for calculation of immune infiltration

To see if altered ccRCC secretome could affect TME, we next analyzed the correlations between the expression of the 85 genes encoding proteins of ccRCC secretome and the immune infiltration. The expression of genes encoding top-altered secretome proteins, including (SPARC, INHBA) highly correlated (r > 0.7) with the presence of endothelial cells and CAFs, respectively (Additional file 1: Table S8, Fig. 4). Furthermore, the term for angiogenesis processes in GO analysis results was enriched with the genes whose expression correlated with SPARC in ccRCC tumors (Fig. 4B, Additional file 1: Table S9). In particular, the expression SPARC was highly correlated with JAM3 (r = 0.81) and RHOJ (r = 0.80), two important promoters of tumorous angiogenesis [29, 30]. Furthermore, the genes whose expression correlated with SPARC in ccRCC tumors, were enriched in the molecular functions associated with vessel growth such as “Platelet-derived growth factor binding” (Additional file 1: Table S9).

Next, to see if the genes of ccRCC secretome could be involved in TME shaping in other cancer types, we repeated the analysis of the association of 85 secretome genes with immune infiltration in 40 cancer types and > 12,000 tumor samples. Strikingly, SPARC emerged as the top gene (r ≥ 0.9) correlating with CAFs across various cancer types (Additional file 1: Table S10, Fig. 4), while its expression was commonly upregulated across different tumors, including cancer of bladder, breast, bile ducts, colon, esophagus, brain, head and neck, liver, and stomach (Additional file 2: Fig. S3). The genes positively correlating with SPARC in breast and colon adenocarcinoma tumors included TGFB1, a powerful stimulator of migration of peritumoral CAFs [31]. Notably, TGF-β cooperates with PDGF to regulate CAFs differentiation [32] and “Platelet-derived growth factor binding” was the second top-enriched molecular function in the genes correlating with SPARC in breast and colon cancers (Additional file 2: Table S9).

### Metabolome analysis reveals changes in ccRCC conditioned media

**Fig. 5:**
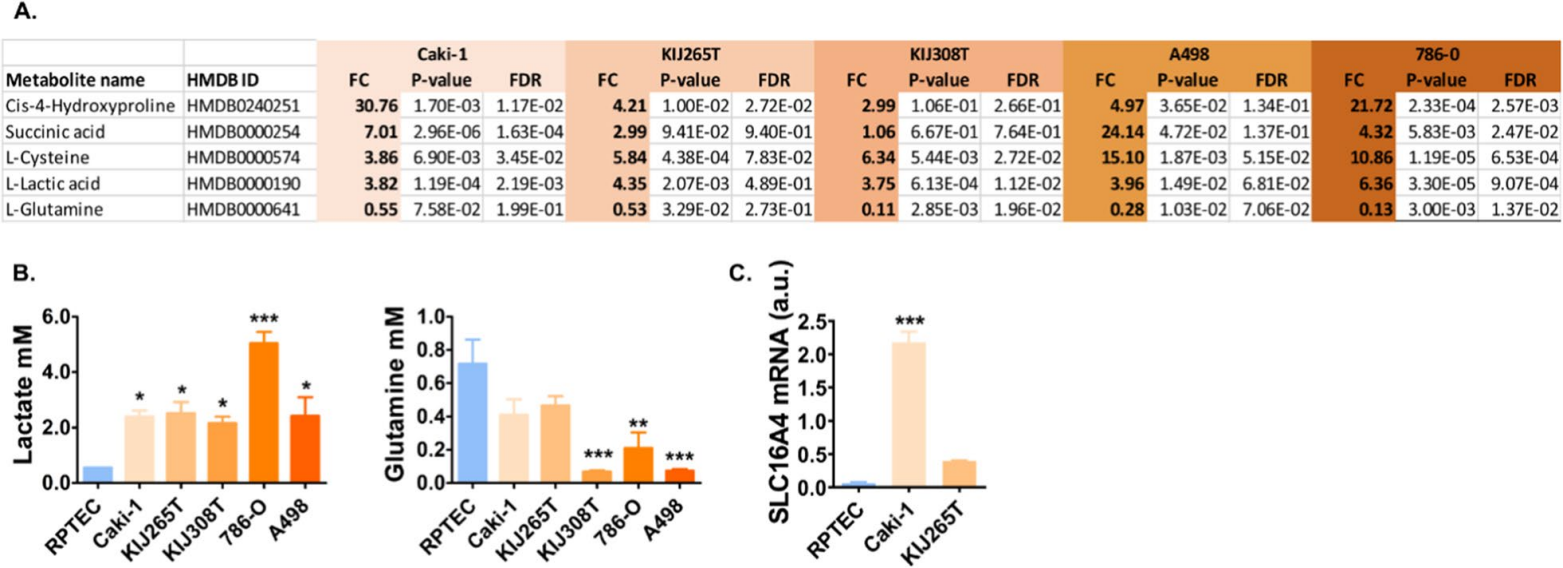
ccRCC CM metabolome changes. **A** Top altered CM metabolites analyzed by GC–MS (complete GC–MS data are shown in Additional file 2: Table S11). **B** Validation of GC–MS. **C** The expression of SLC16A4 lactate transporter in ccRCC cells. The plot shows results of qPCR validation of microarray data. The analyses were performed using conditioned media (A, B) or RNA (C) isolated from 3 independent biological experiments. Statistical analysis: One-way ANOVA with Dunnett's Multiple Comparison Test. *p < 0.05, **p < 0.01, ***p < 0.001

GC–MS analysis showed that the top altered CM metabolites included increased 4-hydroxy-proline, succinic acid, L-cysteine, L-lactic acid, as well as downregulated glutamine (Additional file 2: Table S11). Metabolite validation analysis confirmed uniformly upregulated lactate and downregulated glutamine (Fig. 5). Cysteine and succinate were on the border of detection limit (not shown). We did not find a reliable assay to verify 4-hydroxy-proline levels.

To search for the mechanisms behind changes in ccRCC CM metabolome, we looked into the transcriptomes of ccRCC cells. The expression of 34 genes encoding solute carrier proteins (SLC) was commonly changed in KIJ265T and Caki-1 when compared with RPTEC (Additional file 2: Table S5). The top upregulated SLC gene was SLC16A4 (MCT4) encoding lactate transporter. qPCR confirmed upregulated SLC16A4 in Caki-1 and KIJ265T cells (Fig. 5).

## Discussion

The tumor secretome plays a crucial role in shaping TME. We identified 85 proteins that are the constituents of ccRCC secretome and confirmed components of human plasma. We show that transcriptomic changes in cancer cells reflect their altered secretome, not only by means of altered expression of genes encoding the key secreted proteins, but also by changes in genes involved in protein transport, secretion, and export. Immune infiltration correlates with top altered genes encoding ccRCC secretome, of which SPARC emerges as a PanCancer indicator of CAFs infiltration. Finally, we found that secretome changes are associated with altered concentrations of secreted metabolites, including upregulated lactate and reduced glutamine.

The components of ccRCC secretome are known regulators of TME and cancer cells. SPARC (secreted protein acidic cysteine-rich) is a multifunctional matricellular glycoprotein, contributing to cancerous angiogenesis, EMT, and immune suppression [42, 43]. In renal cancer, SPARC mediates TGF-β-induced metastasis by facilitating cancerous invasion [44]. Our study indicates that high SPARC expression correlates with the presence of endothelial cells in ccRCC tumors. This fits previous reports showing that SPARC contributes to angiogenesis by inducing migration of pericytes, the cells which coat nascent endothelial tubes during vessel formation [45] and induces endothelial permeability to facilitate metastasis [46]. SPARC induces TGFBI deposition [47] which is reflected by our data showing increased TGFBI amounts in ccRCC secretome. SPARC’s role in ccRCC may exceed the association with angiogenesis. ccRCC is highly related to obesity and the tumors are enriched in fatty tissue [48]. SPARC induces recruitment of adipose stem cells, required for fat expansion [49]. Thus, it is tempting to speculate that high-fat content in ccRCC tumors may be associated with enhanced SPARC secretion; this hypothesis requires experimental verification. All these results suggest that increased SPARC secretion by ccRCC cells may result in autocrine and paracrine mechanisms facilitating invasion and affecting TME.

Among the 85 proteins of ccRCC secretome, SPARC emerged as a Pan-cancer indicator of CAFs tumor infiltration. CAFs contribute to ECM remodeling, angiogenesis, and immune suppression [50]. High CAFs infiltration correlates with poor prognosis for cancer patients [50]. Here, high SPARC-CAFs correlations were found for 18 cancer types (Fig. 4). CAFs originate from different types of cells, including pericytes, adipocytes or endothelial cells [50]. Thus, it may be hypothesized that high CAFs content in certain tumors may result from the SPARC-induced recruitment of precursory cells which differentiate into CAFs. The specific mechanisms by which SPARC may induce CAFs accumulation in tumors require further exploration. Apart from SPARC, the other genes correlating with immune infiltration included SPOCK1 and INHBA which were highly correlated with CAFs in colon cancer, as well as TUBB (correlations with myeloid dendritic cells in thymoma) (Additional file 2: Table S10). All these genes are potential biomarkers of immune infiltrations and promising targets for future therapies targeting TME.

SERPINE1 (plasminogen activator inhibitor-1, PAI-1) was one of the most abundant proteins in ccRCC secretome, with concentrations exceeding 25 ng/ml in CM from Caki-1 and 786-O cell lines (Fig. 1). In contrast, there was only minimal secretion of SERPINE1 in RPTEC cells. SERPINE1 is a well-known component of human secretome involved in senescence, fibrosis, and cancerous progression [51–53], released by cancer cells [52, 54–56] and TME components including adipocytes, osteoblasts, CAFs, and MSCs [57–61]. High SERPINE1 expression correlates with the immune infiltration in ccRCC tumors [62] and poor prognosis for ccRCC patients [63]. SERPINE1 acts in an autocrine manner, contributing to the motility of ccRCC cells [64].

TGFBI (BIGH3) is a well-known component of ECM, secreted by multiple cells of tumors and TME [65–67], contributing to angiogenesis [66], immunosuppression [68], ECM adhesion [69], and extravasation [70]. Here, TGFBI was consistently upregulated in CM from ccRCC cells, with the highest amounts detected in 786-O and A498 cells (Fig. 1). This is in agreement with a previous study showing that TGFBI secreted by 786-O cells promotes formation of osteolytic lesions [7]. CM from 786-O cells contained also the highest concentration of peroxiredoxin (PRDX2) which contributes to the bone metastasis by inducing pathological bone destruction resulting from osteoclast activity [71]. This suggests that 786-O cells may be particularly predisposed to the formation of bone metastasis due to enhanced secretion of PRDX2 and TGFBI, two proteins inducing bone lesions. PRDX2 belongs to the family of peroxiredoxins, the enzymes involved in antioxidative responses. PRDX2 is secreted by various cancer cells, including cells of cervical cancer, erythroleukemia, lung adenocarcinoma [71–73] as well as macrophages and embryonic kidney cells [74]. It stimulates the release of TNFa by macrophages and was suggested to modulate immunity by influencing disulfide-mediated dimerization of cytokines [74]. In our study, the ccRCC-derived cell lines released variable amounts of PRDX2, despite consistently decreased mRNA expression in ccRCC cell lines and tumor tissues (Figs. 1 and 2). In contrast, the data from the CPTAC consortium showed increased PRDX2 protein in ccRCC tumors (Fig. 2). To some extent, these data are consistent with previous study that showed that macrophages and embryonic kidney cells release PRDX2 in response to extracellular stimulus (e.g. LPS or TNFa), while the increase in extracellular PRDX2 concentration is not associated with the upregulation of mRNA transcript [74]. Other studies suggested that PRDX2 is mainly controlled at the proteasomal level [75]. Moreover, ccRCC tumors are highly heterogenic and other, non-cancerous cells may contribute to the level of PRDX2. In particular, ccRCC tumors are highly infiltrated by T cells [76] which express PRDX2 [77]. Therefore, the final detectable amount of PRDX2 in tumors may depend on the net effect of the presence of non-cancer cells infiltrating the tumor.

Transferrin (TF) and DPP7 were among proteins consistently suppressed in ccRCC CM. Transferrin cooperates with its receptors (TFR) to deliver iron into the cells. TF/TFR complex is internalized, with the following iron release in the endosomes [78]. Next, TF is directed for either degradation or recycling, preferably by the latter pathway. ccRCC is highly dependent on iron. Tumor-associated macrophages (TAMs) provide iron into ccRCC cells, thereby supporting their proliferation and migration [79]. The expression of TFRC is enhanced in ccRCC tumors and correlates with cancer progression [80]. Therefore, the loss of TF from ccRCC-conditioned media reflects its enhanced uptake mediated by TFRC. DPP7 (DPP2) is a soluble N-terminal dipeptidase, localizing to the cellular vesicles and secreted in a Ca^2+^-regulated manner [81]. The role of DPP7 in cancers, including ccRCC, is largely unknown. The key function of kidney peptidases is catabolism of reabsorbed peptides [82]. Thus, the loss of DPP7 could potentially lead to the increased concentrations of its uncleaved substrates. Indeed, our MS analysis revealed increased 4-hydroxy-proline in CM from ccRCC cells, while hydroxyproline is a substrate of DPP7 [82]. However, if increased 4-hydroxyproline concentrations result from the loss of DPP7 from ccRCC secretome, needs to be experimentally verified.

In our study, the two most altered extracellular metabolites were reduced glutamine and upregulated lactate. This reflects the well-known metabolic ccRCC reprogramming defined by Warburg effect and high glutamine consumption. The changes in extracellular metabolites can affect TME. High glutamine consumption by ccRCC cells leads to the local glutamine deprivation which in turn triggers IL-23 secretion by TAMs [83]. High lactate secretion and enhanced expression of SLC16A4 transporter in ccRCC cells are in line with previous reports [84, 85]. Lactate is a powerful regulator of cancer progression. Specifically, lactate secreted by cancer cells and CAFs may serve as a fuel for cancer cells when glucose is limited. Cancer-secreted lactate leads to TME acidosis, facilitating metastasis, angiogenesis and immunosuppression. Lactate induces apoptosis and attenuates proliferation of immune cells [6]. GC–MS analysis of CM from ccRCC cells showed increased extracellular levels of succinate, an important regulator of TME which drives metastasis by enhancing migration and/or invasion of macrophages and cancer cells [86]. The mechanism of succinate secretion by cancer cells is unknown; therefore, the causes of the increased extracellular succinate concentrations in ccRCC require further exploration.

**Fig. 6:**
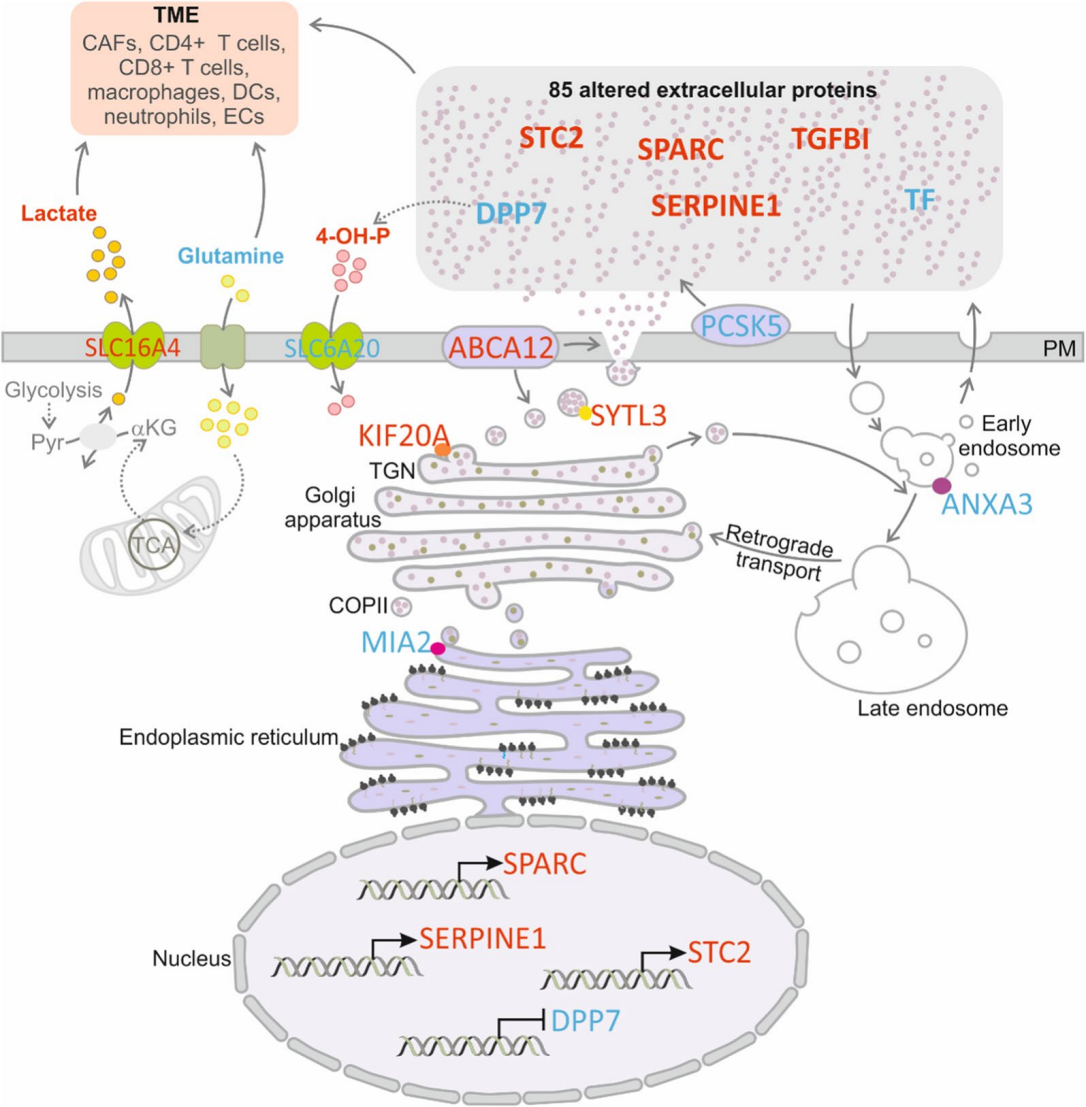
Coordinated reprogramming of ccRCC transcriptome, metabolome and secretome. The expression of genes encoding SPARC, SERPINE1 and STC2 is upregulated while expression of DPP7 is downregulated in ccRCC cells, which reflects altered levels of the encoded protein in the extracellular milieu. The expression of genes encoding proteins involved in protein trafficking and secretion is reprogrammed to support changes in concentrations of extracellular proteins and metabolites: MIA2 (cTAGE5) a receptor of endoplasmic reticulum, localizing to the ER exit sites (ERES), a critical regulator of COPII assembly, protein trafficking and export [33–36]. ANXA3 localizes to endocytic compartments in ccRCC cells [37] and contributes to the regulation of vesicles release [38]. KIF20A is a kinesin crucial for the fission of RAB6-positive vesicles and their exit from Golgi/TGN membranes [27]. It also promotes secretion of factors involved in proliferation of castration-resistant prostate cancer [39]. SYTL3 is a critical effector of RAB27B, enabling kinesin-microtubule-dependent movement of secretory granules towards plasma membrane [40]. ABCA12 is a transmembrane lipid transporter, required for the proper transcriptional programming of vesicle trafficking and cytoskeletal remodeling pathways, lipid raft composition, as well as formation and functioning of secretory granules in pancreatic cells [41]. PCSK5 is a proprotein convertase, which cleaves the target proproteins converting them into their active functional forms. Enhanced expression of SLC16A4 transporter ensures increased secretion of lactate. Changes in concentrations of extracellular proteins (STC2, SPARC, SERPINE1, TGFBI, DPP7, and TF) as well as metabolites affect the functioning of TME cells. TGN: trans-Golgi network; COPII: The Coat Protein Complex II vesicles; Pyr: pyruvate; αKG: α-keto glutarate; PM: plasma membrane; CAFs: cancer-associated fibroblasts; DCs: dendritic cells; ECs: endothelial cells. 4-OH-P: 4-hydroxyproline. Upregulation/downregulation is shown with red/blue font, respectively

Our study shows reprogramming of the ccRCC transcriptome to support altered secretion of proteins and metabolites (Fig. 6). Upregulated SLC16A4 expression was consistent with increased extracellular lactate, while high extracellular 4-hydroxy-proline levels reflected the reduced expression of its importer, SLC6A20. SLC6A20 expression was decreased in renal tumors (Additional file 2: Fig. S4), suggestive of its clinical importance. Increased extracellular 4-hydroxy-proline may also result from the enhanced degradation of ECM collagens by MMP1 collagenase, highly expressed by ccRCC cells. Consistently with their upregulated secretion, the expressions of SPARC, SERPINE1 and STC2 were increased in ccRCC cells, while decreased extracellular DPP7 concentration was reflected by lowered expression of its encoding gene. Altogether, there were 13 secreted proteins with consistently altered gene expression in ccRCC cells. The changes ccRCC secretome may be also related to altered expression of genes regulating trafficking and secretion. They included a large group of kinesins required for intracellular transport [87]. The top upregulated was KIF20A, crucial for the fission of RAB6-positive vesicles and their exit from Golgi/TGN membranes [27]. ccRCC cells overexpressed SYTL3, a critical effector of RAB27B which enables kinesin-microtubule-dependent movement of secretory granules towards the plasma membrane [40]. SLC9A3R1, which was upregulated in ccRCC, encodes NHERF1, a multifunctional scaffold protein that regulates trafficking in the kidney [88] and secretion of angiogenic factors [28]. In kidney cells, SLC9A3R1 regulates plasma membrane localization of multidrug resistance protein MRP4 [89], which suggests that SLC9A3R1 may be involved in drug efflux in renal tumors. Differential SLC9A3R1 phosphorylation determines metastatic organotropism of cancer cells [28]. SLC9A3R1 was aberrantly phosphorylated in ccRCC tumors, suggesting a potential link with metastasis. The other altered genes included ABCA12, a transmembrane lipid transporter, required for the proper vesicle trafficking and functioning of secretory granules in cancer cells [41] or ANXA3 which affects the expression of cytokine genes and tumor immune infiltration [90]. ANXA3 localizes to the endocytic compartments in ccRCC cells and may interfere with vesicular trafficking, thereby negatively modulating intracellular lipid storage [37]. We also found reduced expression of MIA2, involved in protein trafficking and export [33, 34]. MIA2 regulates the COPII assembly and collagen secretion [35, 36], while its depletion in hepatoma cells leads to the accumulation of lipid droplets [35]. This suggests that downregulation of ANXA3 and MIA2 in ccRCC cells may contribute to the specific fatty phenotype of ccRCC tumors.

## Conclusions

Collectively, our study shows coordinated metabo-/proteo-/transcriptomic reprogramming of ccRCC cells, leading to the changes in the extracellular milieu. Notably, the consistently altered extracellular proteins and metabolites are well-known regulators of TME cells, including macrophages, CAFs, and endothelial cells. Some of the identified proteins (e.g. B2M, SPARC) were already reported in serum or urine of ccRCC patients [91, 92]. This confirms the validity of our study and indicates that the list of the identified 85 proteins is a valuable source of the future serum/urine-based ccRCC biomarkers.

## Supplementary Information

**Additional file 1: Table S1.** The conditions of ELISA assays. **Table S2.** Sequences of primers used for qPCR reactions. **Table S3.** The characteristics of tissue samples used for RNA isolation. N.D.: not determined. **Table S4.** The result of proteomic analysis of conditioned media isolated from five ccRCC-derived cell lines: Caki-1, KIJ265T, KIJ308T, A498, and 786–0, compared with conditioned media isolated from RPTEC cell line, derived from normal proximal tubules. **Table S5.** The results of microarray transcriptomic analysis of ccRCC-derived cell lines (Caki-1, KIJ265T) compared with RPTEC cell line, derived from normal proximal tubules. **Table S6.** The result of GO enrichment analysis of genes commonly altered in Caki-1 and KIJ265T cells when compared with RPTEC. Top enriched GO terms are shown. The analysis was performed using ShinyGO 0.76 (http://bioinformatics.sdstate.edu/go/). **Table S7.** The expression of genes involved in secretion and trafficking in ccRCC tumors. The table shows gene expression in TCGA data, KIRC cohort, analyzed using ENCORI platform (https://starbase.sysu.edu.cn/). **Table S8.** Correlations between the expression of the 85 genes encoding proteins of ccRCC secretome and the immune infiltration in ccRCC tumors. The analysis was performed using http://timer.comp-genomics.org/. **Table S9.** GO enrichment analysis of genes of which expression correlates with SPARC in KIRC, BRCA, and COAD TCGA data. The list of genes correlating with SPARC in KIRC, BRCA, and COAD tumors was generated using UALCAN platform. GO analysis was performed using ShinyGO (http://bioinformatics.sdstate.edu/go/). **Table S10.** The association of 85 secretome genes with immune infiltration in 40 cancer types and > 12,000 tumor samples. The analysis was performed using http://timer.comp-genomics.org/. **Table S11.** The results of GC–MS analysis of ccRCC-conditioned media. The table shows metabolites altered in CM from five RCC-derived cell lines when compared with RPTEC.

**Additional file 2: Figure S1**. STRING interaction network of the 85 proteins of ccRCC secretome. Network nodes represent proteins; Edges represent protein–protein associations. **Figure S2.** The ELISA validation of HSP27 and SCIN. The plots show results of three independent experiments. Statistical analysis: One-way ANOVA with Dunnett’s Multiple Comparison Test. **Figure S3.** SPARC expression is commonly upregulated in different cancer types. The plot was generated using Timer (http://timer.comp-genomics.org/). Statistical analysis was performed using Wilcoxon test. *p-value < 0.05; **p-value < 0.01; ***p-value < 0.001. **Figure S4.** Altered expression of SLC6A20 in renal cancer. The plots show results of UALCAN/CPTAC analysis. N: normal kidney samples (n = 84), T: ccRCC tumor samples (n = 110).

## Data Availability

All data supporting the findings of this study are available within the paper. The raw data that support the findings of this study are available from the corresponding author upon reasonable request. The mass spectrometry data were deposited to the ProteomeXchange Consortium [93] via the MassIVE repository with the dataset identifier PXD030085.

## Abbreviations

4-OH-P: 4-Hydroxyproline
ATCC: American Type Culture Collection
CAFs: Cancer-associated fibroblasts
CAFs: Cancer-associated fibroblasts
ccRCC: Clear cell renal cell carcinoma
CM: Conditioned media
COPII: The Coat Protein Complex II vesicles
DCs: Dendritic cells
ECM: Extracellular matrix
ECs: Endothelial cells
ELISA: Enzyme-linked immunosorbent assay
KIRC: TCGA-clear cell renal cell carcinoma
mccRCC: Metastatic ccRCC
PM: Plasma membrane
PMNs: Pre-metastatic niches
Pyr: Pyruvate
qPCR: Quantitative real-time PCR
RCC: Renal cell carcinoma
SLC: Solute carrier
SPARC: Secreted protein acidic cysteine-rich
TCGA: The Cancer Genome Atlas
TGN: Trans-Golgi network
TME: Microenvironment
αKG: α-Keto glutarate
