# Supplementary material for: Coordinated reprogramming of renal cancer transcriptome, metabolome and secretome associates with immune tumor infiltration
Source: Cancer Cell Int. 2023 Jan 5;23:2. doi: 10.1186/s12935-022-02845-y (PMC9814214; doi:10.1186/s12935-022-02845-y)
Supplement: Supplementary file 1 — Additional file 1: Table S1. The conditions of ELISA assays. Table S2. Sequences of primers used for qPCR reactions. Table S3. The characteristics of tissue samples used for RNA isolation. N.D.: not determined. Table S4. The result of proteomic analysis of conditioned media isolated from five ccRCC-derived cell lines: Caki-1, KIJ265T, KIJ308T, A498, and 786–0, compared with conditioned media isolated from RPTEC cell line, derived from normal proximal tubules. Table S5. The results of microarray transcriptomic analysis of ccRCC-derived cell lines (Caki-1, KIJ265T) compared with RPTEC cell line, derived from normal proximal tubules. Table S6. The result of GO enrichment analysis of genes commonly altered in Caki-1 and KIJ265T cells when compared with RPTEC. Top enriched GO terms are shown. The analysis was performed using ShinyGO 0.76 (http://bioinformatics.sdstate.edu/go/). Table S7. The expression of genes involved in secretion and trafficking in ccRCC tumors. The table shows gene expression in TCGA data, KIRC cohort, analyzed using ENCORI platform (https://starbase.sysu.edu.cn/). Table S8. Correlations between the expression of the 85 genes encoding proteins of ccRCC secretome and the immune infiltration in ccRCC tumors. The analysis was performed using http://timer.comp-genomics.org/. Table S9. GO enrichment analysis of genes of which expression correlates with SPARC in KIRC, BRCA, and COAD TCGA data. The list of genes correlating with SPARC in KIRC, BRCA, and COAD tumors was generated using UALCAN platform. GO analysis was performed using ShinyGO (http://bioinformatics.sdstate.edu/go/). Table S10. The association of 85 secretome genes with immune infiltration in 40 cancer types and > 12,000 tumor samples. The analysis was performed using http://timer.comp-genomics.org/. Table S11. The results of GC–MS analysis of ccRCC-conditioned media. The table shows metabolites altered in CM from five RCC-derived cell lines when compared with RPTEC. [file 12935_2022_2845_MOESM1_ESM.zip › Supplementary Data/Table S3_ESM.docx]

**Table S3. The characteristics of tissue samples used for RNA isolation.** N.D.: not determined.

| **Patient no.** | **TNM stage** | **Fuhrman grade** |
| --- | --- | --- |
| 1 | T3 | G3 |
| 2 | T3 | G3 |
| 3 | T1 | G2 |
| 4 | T2 | G2 |
| 5 | T2 | G2 |
| 6 | T3 | G2 |
| 7 | T3 | G2 |
| 8 | T2 | G1 |
| 9 | T1 | G1 |
| 10 | T1 | G1 |
| 11 | T1 | G2 |
| 12 | T4 | G1 |
| 13 | T3 | G2 |
| 14 | T3 | G2 |
| 15 | T2 | G2 |
| 16 | T1 | G1 |
| 17 | T1 | G2 |
| 18 | T1 | G2 |
| 19 | T2 | G2 |
| 20 | T4 | G3 |
| 21 | T3 | G3 |
| 22 | T1 | G1 |
| 23 | T3 | G2 |
| 24 | T1 | G1 |
| 25 | T3 | G1 |
| 26 | T2 | G3 |
| 27 | T1 | G1 |
| 28 | T3 | G2 |
| 29 | T1 | G2 |
| 30 | T3 | G2 |
| 31 | T1 | G2 |
| 32 | T1 | G2 |
| 33 | T1 | G2 |
| 34 | T1 | G2 |
| 35 | T1 | G2 |
| 36 | T1 | G1 |
| 37 | T3 | G3 |
| 38 | T1 | G1 |
| 39 | T3 | G2 |
| 40 | T3 | G2 |
| 41 | T2 | G2 |
| 42 | T2 | G2 |
| 43 | T2 | G1 |
| 44 | T1 | G1 |
| 45 | T1 | G1 |
| 46 | T2 | G2 |
| 47 | T1 | G1 |
| 48 | T1 | G2 |
| 49 | T3 | G3 |
| 50 | T1 | G1 |
| 51 | T1 | G2 |
| 52 | T1 | G2 |
| 53 | T1 | G1 |
| 54 | T3 | G2 |
| 55 | T1 | G2 |
| 56 | T1 | G1 |
| 57 | T1 | G1 |
| 58 | T1 | G1 |
| 59 | T3 | G2 |
| 60 | T1 | G2 |
| 61 | T1 | G1 |
| 62 | T1 | G1 |
| 63 | T1 | G1 |
| 64 | T1 | G2 |
| 65 | T1 | G2 |
| 66 | T1 | G2 |
| 67 | T3 | G2 |
| 68 | T1 | G2 |
| 69 | T3 | G3 |
| 70 | T3 | G2 |
| 71 | T4 | N.D. |
| 72 | T1 | G1 |
| 73 | T3 | G2 |
| 74 | T3 | G2 |
| 75 | T1 | G1 |
| 76 | T3 | G2 |
| 77 | T2 | G2 |
| 78 | T3 | G2 |
| 79 | T3 | G2 |
| 80 | T1 | G2 |
| 81 | T3 | G2 |
| 82 | T1 | G1 |
| 83 | T1 | G2 |
| 84 | T2 | G4 |
| 85 | T1 | G3 |
| 86 | T3 | G2 |
| 87 | T1 | G2 |
| 88 | T1 | G1 |
| 89 | T1 | N.D. |
| 90 | T1 | N.D. |
| 91 | T1 | N.D. |
| 92 | T4 | N.D. |
