# Supplementary material for: Coordinated reprogramming of renal cancer transcriptome, metabolome and secretome associates with immune tumor infiltration
Source: Cancer Cell Int. 2023 Jan 5;23:2. doi: 10.1186/s12935-022-02845-y (PMC9814214; doi:10.1186/s12935-022-02845-y)
Supplement: Supplementary file 2 — Additional file 2: Figure S1. STRING interaction network of the 85 proteins of ccRCC secretome. Network nodes represent proteins; Edges represent protein–protein associations. Figure S2. The ELISA validation of HSP27 and SCIN. The plots show results of three independent experiments. Statistical analysis: One-way ANOVA with Dunnett’s Multiple Comparison Test. Figure S3. SPARC expression is commonly upregulated in different cancer types. The plot was generated using Timer (http://timer.comp-genomics.org/). Statistical analysis was performed using Wilcoxon test. *p-value < 0.05; **p-value < 0.01; ***p-value < 0.001. Figure S4. Altered expression of SLC6A20 in renal cancer. The plots show results of UALCAN/CPTAC analysis. N: normal kidney samples (n = 84), T: ccRCC tumor samples (n = 110). [file 12935_2022_2845_MOESM2_ESM.docx]

**Additional file 2: Figures**


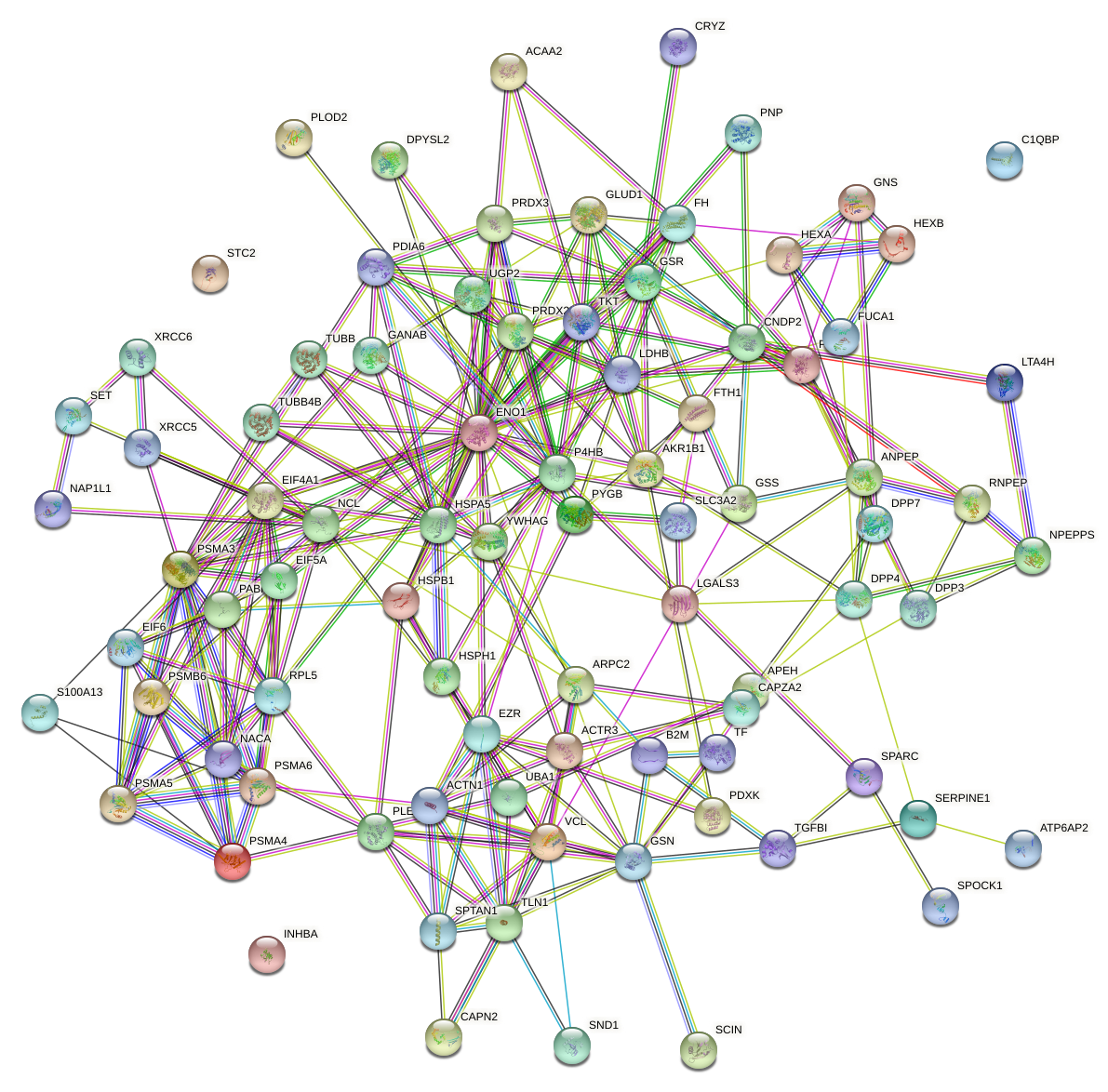


**Figure S1**. STRING interaction network of the 85 proteins of ccRCC secretome. Network nodes represent proteins; Edges represent protein-protein associations.


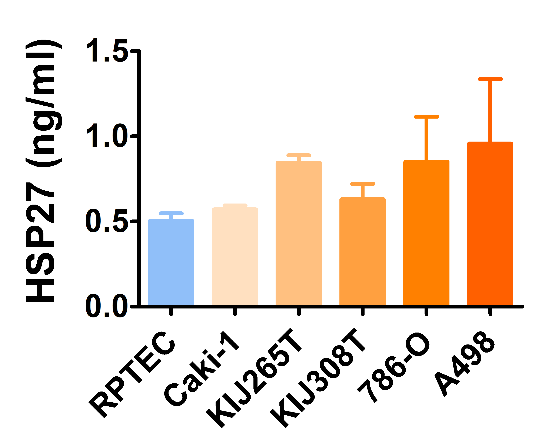

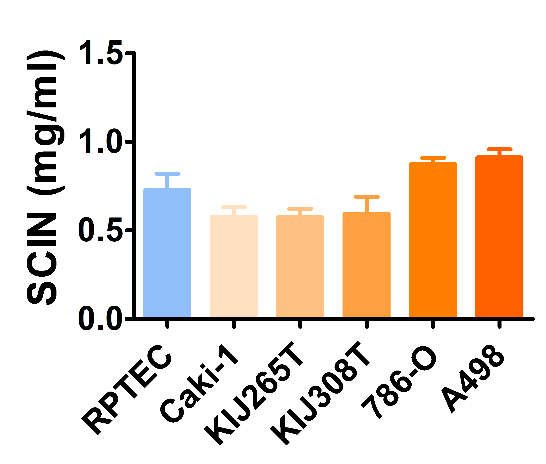


**Figure S2.** The ELISA validation of HSP27 and SCIN. The plots show results of three independent experiments. Statistical analysis: One-way ANOVA with Dunnett's Multiple Comparison Test.


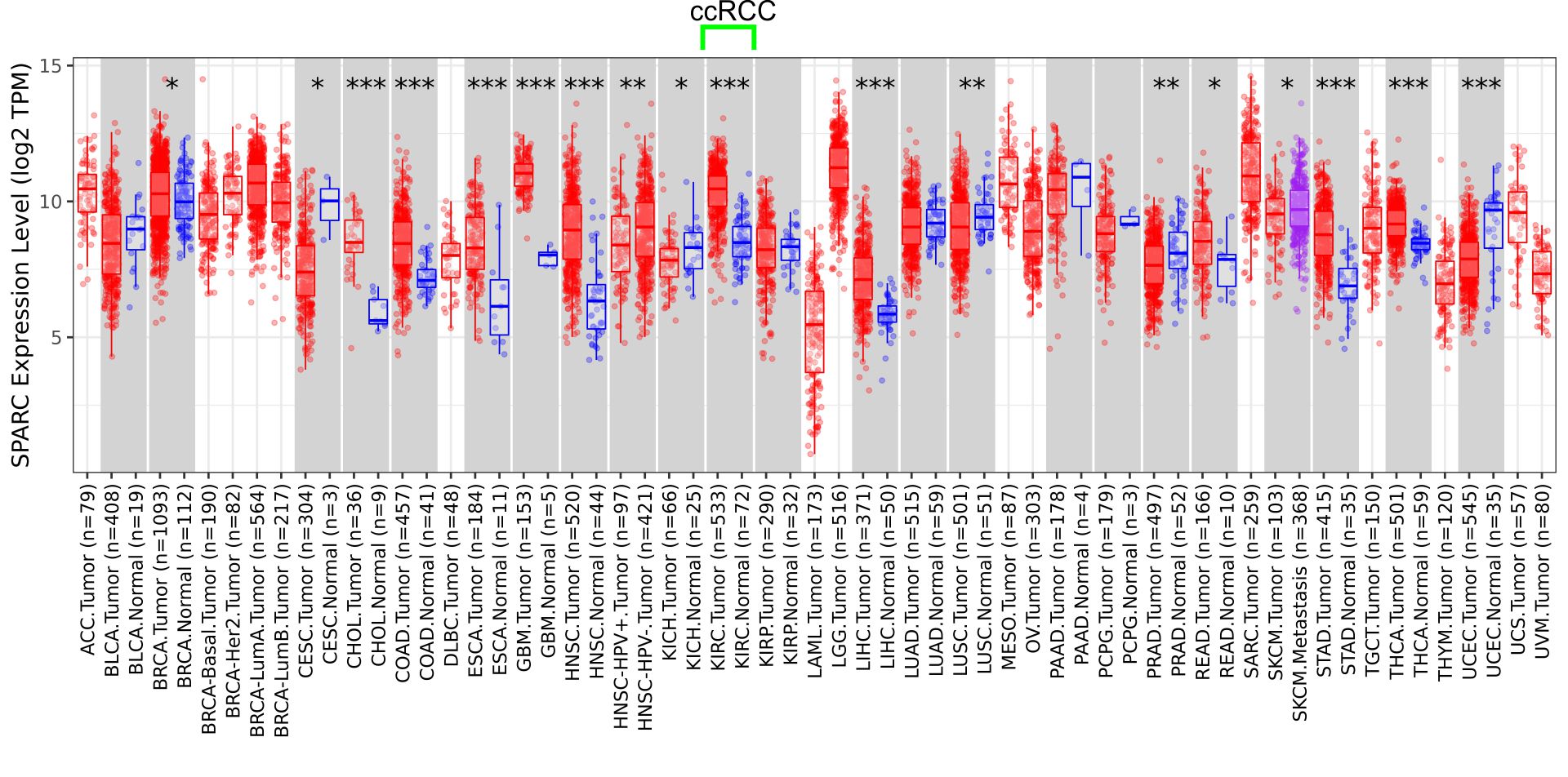


**Figure S3.** SPARC expression is commonly upregulated in different cancer types. The plot was generated using Timer (http://timer.comp-genomics.org/). Statistical analysis was performed using Wilcoxon test. *: p-value < 0.05; **: p-value < 0.01; ***: p-value < 0.001.


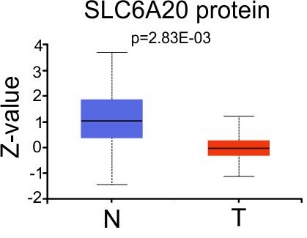


**Figure S4**. Altered expression of SLC6A20 in renal cancer. The plots show results of UALCAN/CPTAC analysis. N: normal kidney samples (n=84), T: ccRCC tumor samples (n=110).
